# Nutrition and Nonalcoholic Fatty Liver Disease: The Significance of Cholesterol

**DOI:** 10.1155/2012/925807

**Published:** 2012-04-05

**Authors:** Munechika Enjoji, Kenichiro Yasutake, Motoyuki Kohjima, Makoto Nakamuta

**Affiliations:** ^1^Health Care Center, Fukuoka University, 8-19-1 Nanakuma, Jonan-ku, Fukuoka 814-0180, Japan; ^2^Clinical Research Center, Kyushu Medical Center, National Hospital Organization, Fukuoka 810-8563, Japan; ^3^Department of Gastroenterology, Kyushu Medical Center, National Hospital Organization, Fukuoka 810-8563, Japan

## Abstract

Nonalcoholic fatty liver disease (NAFLD) is a common chronic liver disease that ranges in severity from simple steatosis to cirrhosis. NAFLD is considered to be associated with hepatic metabolic disorders, resulting in overaccumulation of fatty acids/triglycerides and cholesterol. The pathogenesis and progression of NAFLD are generally explained by the “two-hit theory.” Most studies of lipid metabolism in the NAFLD liver have focused on the metabolism of fatty acids/triglycerides; therefore, the impact of cholesterol metabolism is still ambiguous. In this paper, we review recent studies on NAFLD from the viewpoint of hepatic lipid metabolism-associated factors and discuss the impact of disordered cholesterol metabolism in the etiology of NAFLD. The clinical significance of managing cholesterol metabolism, an option for the treatment of NAFLD, is also discussed.

## 1. Introduction

Histological features of nonalcoholic fatty liver disease (NAFLD) include steatosis, hepatocellular ballooning, the formation of Mallory bodies, apoptosis/necrosis, and inflammation [1]. Around 10–20% of patients with NAFLD have nonalcoholic steatohepatitis (NASH), which can develop into cirrhosis and hepatocellular carcinoma [2–5]. Because excess nutrition intake is one of the main causes, NAFLD is often accompanied by obesity, insulin resistance, hypertension, and/or dyslipidemia, which are manifestations of the metabolic syndrome [6]. Therefore, nutritional management and therapeutic exercise are fundamental steps to treat NAFLD.

The “two-hit theory” is increasingly being adopted to explain the pathogenesis of NAFLD and NASH [7]. In this theory, the first hit consists of the accumulation of fatty acids/triglycerides in the liver, while the second hit involves oxidative stress, mitochondrial dysfunction, and inflammation, which ultimately cause liver damage. It is also clear that inflammatory cytokines and insulin resistance are closely associated with fatty liver during the progression of NAFLD. In previous studies that examined lipid metabolism in the context of NAFLD, dysregulation of cholesterol metabolism has received much less attention than have fatty acids and triglycerides. In this paper, we focus on the role of cholesterol and its metabolites on the pathogenesis of NAFLD, and also the validity of cholesterol management as a method of treating this disease.

## 2. Fatty Acid Metabolism in the NAFLD Liver

Hepatic lipid homeostasis represents a balance between lipid uptake, synthesis, catabolism, and secretion. Therefore, steatosis, a typical characteristic of NAFLD, is expected to be caused by disordered lipid metabolism, particularly inhibition of fatty acid oxidation and enhanced lipogenesis. Many factors involved in hepatic lipid metabolism pathways have been identified, even though the precise cellular networks are not fully elucidated.

Adiponectin regulates hepatic fatty acid uptake and *de novo* lipogenesis. AMP-activated protein kinase (AMPK) works as a metabolic master switch, and its activity is regulated by adiponectin and tumor necrosis factor-*α* (TNF*α*). Inhibition of AMPK results in the activation of sterol regulatory element-binding protein-1c (SREBP-1c), which upregulates fatty acid synthesis-associated enzymes, such as acetyl-CoA carboxylase (ACC) and fatty acid synthase (FAS). This leads to enhanced fatty acid synthesis and overproduction of triglycerides, ultimately resulting in liver steatosis [8]. Fatty acids are used for *β*-oxidation in mitochondria and peroxisomes under the regulation of peroxisome proliferator-activated receptor-*α* (PPAR*α*). Fatty acids are ligands for PPAR*α*, which transactivates the expression of genes involved in the transport, oxidation, and export of free fatty acids, including carnitine palmitoyltransferase-1 (CPT-1), the rate-limiting enzyme in fatty acid *β*-oxidation.

**Figure 1:**
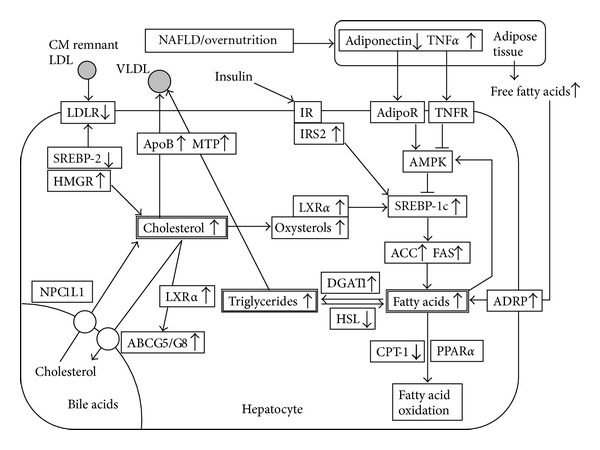
Expression profile of lipid metabolism-associated factors in nonalcoholic fatty liver disease (NAFLD). The established pathophysiological pathways in NAFLD involve increased delivery of fatty acids to the liver and increased SREBP-1c signaling because of cholesterol overload and insulin resistance. ABCG5/G8: ATP-binding cassette G5/G8; ACC: acetyl-CoA carboxylase; AdipoR: adiponectin receptor; ADRP: adipose differentiation-related protein; AMPK: AMP-activated protein kinase; ApoB: apolipoprotein B; CM: chylomicron; CPT-1: carnitine palmitoyltransferase-1; DGAT1: diacylglycerol acyltransferase 1; FAS: fatty acid synthase; HMGR: HMG-CoA reductase; HSL: hormone sensitive lipase; IR: insulin receptor; IRS2: insulin receptor substrate 2; LDLR: LDL receptor; LXR*α*: liver X receptor *α*; MTP: microsomal triglyceride transfer protein; NPC1L1: Niemann-Pick C1-like 1; PPAR*α*: peroxisome proliferator-activated receptor *α*; SREBP: sterol regulatory element-binding protein; TNF*α*: tumor necrosis factor *α*; TNFR: TNF receptor.

The relationship between NAFLD and lipid metabolism has been extensively investigated in studies that analyzed the hepatic gene expression profile in animals fed a high-fat diet [9] and in liver biopsy samples from NAFLD patients [10–14], and their expression profiles have been compared with those in normal individuals.  Figure 1 summarizes the pathological changes in the NAFLD liver, which may lead to the accumulation of triglycerides, free fatty acids, and cholesterol. To our knowledge, the following events are known to occur in NAFLD. First, hepatic steatosis develops because of upregulated fatty acid synthesis, but it is questionable whether downregulation of fatty acid oxidation is also involved [15–18]. Second, adiponectin production is reduced because of increased visceral fat accumulation. Adiponectin levels are inversely proportional to insulin resistance and hepatic steatosis in NAFLD patients [19]. Third, insulin resistance, which is common in NAFLD, causes fatty liver, while increases in hepatocyte fatty acids levels cause hepatic insulin resistance [20]. Fourth, the severity of insulin resistance is correlated with the severity of NASH. Fifth, disturbed insulin signaling in hepatocytes leads to steatosis associated with the activation of SREBP-1c and the induction of fatty acid synthesis [21].

Recent findings suggest that the cannabinoid system is also involved in the development of fatty liver [22–24]. In animal studies, cannabinoid 1 (CB1) receptors were activated by a high-fat diet *via* induction of the synthesis of endocannabinoids, such as 2-arachidonoylglycerol and anandamide. CB1 receptor activation enhanced the expression of several lipogenic factors, including SREBP-1c, ACC and FAS, and downregulated CPT-1, resulting in increased *de novo* fatty acid synthesis and suppression of fatty acid oxidation. However, in the context of lipid metabolism, the signaling pathway downstream of the cannabinoid receptor has not been identified.

## 3. Cholesterol Metabolism in NAFLD

In humans, cholesterol is absorbed from the diet and synthesized by cells in various tissues. A healthy man weighing 60 kg contains approximately 140 g of cholesterol, but only 1% of the total cholesterol is involved in a dynamic metabolic cycle [25]. In one study, the mean intake of dietary cholesterol was estimated to be 300–500 mg/day [14]. They also reported that the dietary cholesterol aggregates into micelles with biliary cholesterol (800–1300 mg/day) in the duodenum [14]. Physiologically, approximately 50% of the cholesterol is absorbed in the jejunum *via* a cholesterol transporter Niemann-Pick C1-like 1 (NPC1L1) expressed on the brush border membrane. The cholesterol is then transported to the liver in the form of chylomicrons and chylomicron remnants [26]. NPC1L1, which may facilitate the hepatic accumulation of cholesterol, is expressed on the canalicular membrane of hepatocytes in humans. Another transporter pump system involving ATP-binding cassette (ABC) G5/G8 excretes cholesterol into bile [27].

The main metabolic pathways of cholesterol in hepatocytes include (1) cholesterol *de novo* synthesis (acetyl-CoA-mevalonate-cholesterol pathway); (2) cholesterol uptake in the form of LDL and chylomicron remnants; (3) cholesterol excretion into the blood in the form of VLDL; (4) cholesterol excretion and uptake through bile *via* ABCG5/G8 and NPC1L1, respectively; (5) synthesis of bile acids and their excretion. Under normal conditions, these pathways interact with each other to maintain cholesterol levels within a specific range.

However, in NAFLD patients, these systems are highly disorganized. SREBPs act as regulators of hepatic cholesterol levels and activate genes involved in the synthesis of cholesterol and free fatty acids [28]. SREBP cleavage-activating protein (SCAP) has a cholesterol-sensing domain that senses intracellular cholesterol levels and directs the activity of SREBPs. Physiologically, when intracellular cholesterol levels are low, SREBPs are first translocated to the Golgi apparatus by SCAP and undergo proteolytic cleavage. Next, the cleaved activated form of SREBP is released to the nucleus. When intracellular cholesterol levels are high, SCAP activity and SREBP activation are suppressed. However, in the context of NAFLD, the regulatory loop of SREBP is disturbed, even if the intracellular levels of cholesterol and/or fatty acids are high [29]. In our study using liver biopsy samples from NAFLD patients, despite excess cholesterol accumulation in hepatocytes, *de novo* cholesterol synthesis remained greatly enhanced even though SREBP-2 expression was downregulated [30]. In the liver of these patients, as evidence of excess cholesterol accumulation, cholesterol uptake was reported to be suppressed because of markedly downregulated expression of LDL receptor (LDLR). Cholesterol excretion was enhanced *via* overexpression of ABCG5/G8, apolipoprotein B, and microsomal triglyceride transfer protein (MTP) [30], but it was considered that the secretion of cholesterol reaches a plateau in NAFLD patients. Even in this situation, cholesterol synthesis continued with upregulated expression of HMG-CoA reductase and synthase, farnesyl P-P synthase and squalene synthase [30–32]. This is because excess levels of cholesterol and its oxysterol metabolites, which are agonists for liver X receptor-*α* (LXR*α*) [32], cause excess fatty acid synthesis and steatosis by activating the LXR*α*-SREBP-1c pathway. LXR*α* expression was also upregulated in the liver of NAFLD patients [31, 32]. As shown in Figure 1, cholesterol uptake in the form of LDL is limited by the intracellular accumulation of fatty acid and cholesterol, while fatty acid and cholesterol synthesis are upregulated in the NAFLD liver. These findings suggest that the feedback system regulating intracellular lipids levels is disrupted in NAFLD.

## 4. Nutritional Analysis in NAFLD Patients

In some nutritional investigations, it has been suggested that high-fat, high-fat plus low-protein, high-carbohydrate, and/or high-cholesterol diets are the main causes of NAFLD [33–36]. Although many NAFLD patients show excess nutrition intake, obese, and/or insulin resistance, not all patients exhibit these features. In our nutritional analysis on Japanese population, nonobese NAFLD patients had some features that differed from those of obese patients [37]. Naturally, the dietary intake levels of total energy, fat, and carbohydrate were markedly higher in obese NAFLD patients with insulin resistance than those in nonobese NAFLD patients and healthy volunteers. The most interesting finding was that cholesterol intake was significantly higher in nonobese NAFLD patients than in obese NAFLD patients although cholesterol intake in obese patients was also significantly higher than that in healthy volunteers. In our hepatic expression analysis of lipid metabolism-associated genes, we found that LXR*α* expression levels were significantly higher in nonobese NAFLD patients than in obese NAFLD patients [13]. Of note, cholesterol overload can upregulate LXR*α* expression and activate fatty acid synthesis by increasing oxysterol levels, metabolites of cholesterol that act as agonists for LXR*α* and activate the LXR*α*-SREBP-1c pathway. These nutrition and gene expression profiles indicate that excess cholesterol intake (i.e., cholesterol supply) itself can be a strong stimulant for the development of steatosis, even though the total calorie intake may be within the normal range. Recent reports using model animals support our findings in nonobese NAFLD patients. Fatty liver without obesity can be established in animal models by feeding them with a hypercholesterolemic diet containing normal calorie level [38–40]. Although this animal model showed marked hypercholesterolemia, which was not observed in our patients, this may be because the diet for animals contains a very high cholesterol content (0.2–1.25%). Moreover, serum cholesterol levels may be preserved in NAFLD patients because dietary cholesterol is promptly taken up into the hepatocyte cholesterol pool.

## 5. Prospects for Cholesterol Management Therapy

As described above, it seems that cholesterol overload initiates the development of NAFLD. The progression from simple steatosis to steatohepatitis (NASH) usually involves the second hit, such as oxidative stress and inflammation. In some studies of nutritional animal models, the accumulation of cholesterol rather than fatty acids/triglycerides plays a critical role in this progression, possibly because of increased susceptibility to oxidative cell death [41]. It has also been suggested that the regulation of cholesterol can control C-reactive protein levels and insulin sensitivity [41]. Conversely, in some reports, the progression of triglyceride accumulation and suppression of fatty acid oxidation were not hepatotoxic and actually protected against worsening liver damage [42]. Therefore, cholesterol management may be a promising treatment target for NAFLD.

Ezetimibe, a blood cholesterol lowering agent, is a NPC1L1-specific inhibitor and selectively blocks 54% of cholesterol absorption from the intestine in humans and in animals [43, 44]. Ezetimibe is quickly absorbed, enters the enterohepatic circulation, and has a half-life of 24 hours. From a nutritional point of view, it is important that ezetimibe does not inhibit the absorption of fat-soluble vitamins. In our clinical study, nonobese NAFLD patients showing excess intake of dietary cholesterol were treated with ezetimibe [45]. After starting the therapy, although significant changes were not seen in their body weight, their serum ALT levels decreased by 49.3 ± 16.1% and 45.3 ± 24.2% at 6 and 12 months, respectively. Moreover, steatotic findings on ultrasonography improved in some patients. Interestingly, NPC1L1 knockout mice with excess nutrition intake were resistant to fatty liver, while ezetimibe elicits therapeutically significant effects in animal models of NAFLD [46, 47]. These findings demonstrate that over intake and hepatic accumulation of cholesterol, leading to the activation of the LXR*α*-SREBP-1c pathway, are closely related to the development of NAFLD. Accordingly, inhibiting cholesterol absorption or reducing dietary cholesterol intake may offer a reliable therapeutic strategy for NAFLD. It has also been reported that HMG-CoA reductase inhibitors (statins) improve serum ALT levels in NAFLD patient [48–50].

Considering these findings, reducing hepatocytic accumulation of cholesterol may represent a fundamental treatment strategy for NAFLD [51]. To establish treatments focusing on cholesterol management, more clinical evidence is clearly needed. For example, cholesterol-restricted diet or lipid modulators (ezetimibe and statins) may be less effective in obese NAFLD patients with insulin resistance than in nonobese patients. This is because other factors associated with obesity and insulin resistance are involved in the development of fatty liver, and these factors may mask the effect of ezetimibe. There are other questions that also need to be answered. For example, does the therapeutic effect of statin in combination with ezetimibe surpass that of monotherapy? Can long-term cholesterol management with ezetimibe and/or statins really improve steatosis as well as ALT levels in NAFLD? It is also important to assess whether the clinical effects of cholesterol management are observed in patients with steatohepatitis (i.e., NASH) as well as patients with simple steatosis, and whether there is a synergistic/additive effect of cholesterol management in combination with antioxidant therapy or liver protection therapy.

## 6. Conclusions

The feedback system controlling intracellular lipids levels is greatly disrupted in NAFLD. Lifestyle modifications offer simple therapeutic targets for NAFLD. Cognitive nutritional support, which is aimed at reducing calorie-intake and avoiding overeating, should be developed alongside pharmaceutical treatments to prevent the disease progression to cirrhosis and HCC. Excess cholesterol intake, in particular, is a major stimulant for the development of fatty liver. The accumulation of cholesterol rather than triglycerides may play a critical role in the progression from simple steatosis to steatohepatitis. Accordingly, strategies targeting cholesterol accumulation offer basic therapeutic approaches for NAFLD patients, and cholesterol management therapy seems to represent a promising treatment for NAFLD. The potential clinical benefit of cholesterol management for treating NAFLD with respect to hepatic steatosis and injury should be estimated in appropriately designed trials.

## References

[1] Brunt EM, Tiniakos DG (2010). Histopathology of nonalcoholic fatty liver disease. *World Journal of Gastroenterology*.

[2] Schaffner F, Thaler HH (1986). Nonalcoholic fatty liver disease. *Progress in Liver Diseases*.

[3] Hashimoto E, Taniai M, Kaneda H (2004). Comparison of hepatocellular carcinoma patients with alcoholic liver disease and nonalcoholic steatohepatitis. *Alcoholism: Clinical and Experimental Research*.

[4] Hashimoto E, Yatsuji S, Tobari M (2009). Hepatocellular carcinoma in patients with nonalcoholic steatohepatitis. *Journal of Gastroenterology*.

[5] Yatsuji S, Hashimoto E, Tobari M, Taniai M, Tokushige K, Shiratori K (2009). Clinical features and outcomes of cirrhosis due to non-alcoholic steatohepatitis compared with cirrhosis caused by chronic hepatitis C. *Journal of Gastroenterology and Hepatology*.

[6] Angulo P (2002). Medical progress: nonalcoholic fatty liver disease. *The New England Journal of Medicine*.

[7] James OF, Day CP (1998). Non-alcoholic steatohepatitis (NASH): a disease of emerging identity and importance. *Journal of Hepatology*.

[8] You M, Matsumoto M, Pacold CM, Cho WK, Crabb DW (2004). The role of AMP-activated protein kinase in the action of ethanol in the liver. *Gastroenterology*.

[9] Xie Z, Li H, Wang K (2010). Analysis of transcriptome and metabolome profiles alterations in fatty liver induced by high-fat diet in rat. *Metabolism*.

[10] Enjoji M, Yada R, Fujino T (2009). The state of cholesterol metabolism in the liver of patients with primary biliary cirrhosis: the role of MDR3 expression. *Hepatology International*.

[11] Higuchi N, Kato M, Shundo Y (2008). Liver X receptor in cooperation with SREBP-1c is a major lipid synthesis regulator in nonalcoholic fatty liver disease. *Hepatology Research*.

[12] Kohjima M, Enjoji M, Higuchi N (2007). Re-evaluation of fatty acid metabolism-related gene expression in nonalcoholic fatty liver disease. *International Journal of Molecular Medicine*.

[13] Nakamuta M, Kohjima M, Higuchi N (2008). The significance of differences in fatty acid metabolism between obese and non-obese patients with non-alcoholic fatty liver disease. *International Journal of Molecular Medicine*.

[14] Nakamuta M, Kohjima M, Morizono S (2005). Evaluation of fatty acid metabolism-related gene expression in nonalcoholic fatty liver disease. *International Journal of Molecular Medicine*.

[15] Browning JD, Horton JD (2004). Molecular mediators of hepatic steatosis and liver injury. *Journal of Clinical Investigation*.

[16] Cheung O, Sanyal AJ (2009). Recent advances in nonalcoholic fatty liver disease. *Current Opinion in Gastroenterology*.

[17] Chalasani N, Gorski JC, Asghar MS (2003). Hepatic cytochrome P450 2E1 activity in nondiabetic patients with nonalcoholic steatohepatitis. *Hepatology*.

[18] Kotronen A, Seppälä-Lindroos A, Vehkavaara S (2009). Liver fat and lipid oxidation in humans. *Liver International*.

[19] Bugianesi E, Gastaldelli A, Vanni E (2005). Insulin resistance in non-diabetic patients with non-alcoholic fatty liver disease: sites and mechanisms. *Diabetologia*.

[20] Savage DB, Semple RK (2010). Recent insights into fatty liver, metabolic dyslipidaemia and their links to insulin resistance. *Current Opinion in Lipidology*.

[21] Chen G, Liang G, Ou J, Goldstein JL, Brown MS (2004). Central role for liver X receptor in insulin-mediated activation of SREBP-1c transcription and stimulation of fatty acid synthesis in liver. *Proceedings of the National Academy of Sciences of the United States of America*.

[22] Osei-Hyiaman D, DePetrillo M, Pacher P (2005). Endocannabinoid activation at hepatic CB1 receptors stimulates fatty acid synthesis and contributes to diet-induced obesity. *Journal of Clinical Investigation*.

[23] Jeong WI, Osei-Hyiaman D, Park O (2008). Paracrine activation of hepatic CB1 receptors by stellate cell-derived endocannabinoids mediates alcoholic fatty liver. *Cell Metabolism*.

[24] Purohit V, Rapaka R, Shurtleff D (2010). Role of cannabinoids in the development of fatty liver (steatosis). *The AAPS Journal*.

[25] Grundy SM, Metzger AL (1972). A physiological method for estimation of hepatic secretion of biliary lipids in man. *Gastroenterology*.

[26] Altmann SW, Davis HR, Zhu LJ (2004). Niemann-pick C1 Like 1 protein is critical for intestinal cholesterol absorption. *Science*.

[27] Graf GA, Li WP, Gerard RD (2002). Coexpression of ATP-binding cassette proteins ABCG5 and ABCG8 permits their transport to the apical surface. *Journal of Clinical Investigation*.

[28] Goldstein JL, DeBose-Boyd RA, Brown MS (2006). Protein sensors for membrane sterols. *Cell*.

[29] Donohue TM (2007). Alcohol-induced steatosis in liver cells. *World Journal of Gastroenterology*.

[30] Nakamuta M, Fujino T, Yada R (2009). Impact of cholesterol metabolism and the LXR*α*-SREBP-1c pathway on nonalcoholic fatty liver disease. *International Journal of Molecular Medicine*.

[31] Sugimoto T, Yamashita S, Ishigami M (2002). Decreased microsomal triglyceride transfer protein activity contributes to initiation of alcoholic liver steatosis in rats. *Journal of Hepatology*.

[32] Zelcer N, Tontonoz P (2006). Liver X receptors as integrators of metabolic and inflammatory signaling. *Journal of Clinical Investigation*.

[33] Musso G, Gambino R, De Michieli F (2003). Dietary habits and their relations to insulin resistance and postprandial lipemia in nonalcoholic steatohepatitis. *Hepatology*.

[34] Solga S, Alkhuraishe AR, Clark JM (2004). Dietary composition and nonalcoholic fatty liver disease. *Digestive Diseases and Sciences*.

[35] Toshimitsu K, Matsuura B, Ohkubo I (2007). Dietary habits and nutrient intake in non-alcoholic steatohepatitis. *Nutrition*.

[36] Thuy S, Ladurner R, Volynets V (2008). Nonalcoholic fatty liver disease in humans is associated with increased plasma endotoxin and plasminogen activator inhibitor 1 concentrations and with fructose intake. *The Journal of Nutrition*.

[37] Yasutake K, Nakamuta M, Shima Y (2009). Nutritional investigation of non-obese patients with non-alcoholic fatty liver disease: the significance of dietary cholesterol. *Scandinavian Journal of Gastroenterology*.

[38] Kainuma M, Fujimoto M, Sekiya N (2006). Cholesterol-fed rabbit as a unique model of nonalcoholic, nonobese, non-insulin-resistant fatty liver disease with characteristic fibrosis. *Journal of Gastroenterology*.

[39] Matsuzawa N, Takamura T, Kurita S (2007). Lipid-induced oxidative stress causes steatohepatitis in mice fed an atherogenic diet. *Hepatology*.

[40] Wouters K, van Gorp PJ, Bieghs V (2008). Dietary cholesterol, rather than liver steatosis, leads to hepatic inflammation in hyperlipidemic mouse models of nonalcoholic steatohepatitis. *Hepatology*.

[41] Fernández A, Colell A, Garcia-Ruiz C, Fernandez-Checa JC (2008). Cholesterol and sphingolipids in alcohol-induced liver injury. *Journal of Gastroenterology and Hepatology*.

[42] Yamaguchi K, Yang L, McCall S (2007). Inhibiting triglyceride synthesis improves hepatic steatosis but exacerbates liver damage and fibrosis in obese mice with nonalcoholic steatohepatitis. *Hepatology*.

[43] Sudhop T, Lütjohann D, Kodal A (2002). Inhibition of intestinal cholesterol absorption by ezetimibe in humans. *Circulation*.

[44] Turley SD, Dietschy JM (2003). Sterol absorption by the small intestine. *Current Opinion in Lipidology*.

[45] Enjoji M, Machida K, Kohjima M (2010). NPC1L1 inhibitor ezetimibe is a reliable therapeutic agent for non-obese patients with nonalcoholic fatty liver disease. *Lipids in Health and Disease*.

[46] Davies JP, Scott C, Oishi K, Liapis A, Ioannou YA (2005). Inactivation of NPC1L1 causes multiple lipid transport defects and protects against diet-induced hypercholesterolemia. *Journal of Biological Chemistry*.

[47] Deushi M, Nomura M, Kawakami A (2007). Ezetimibe improves liver steatosis and insulin resistance in obese rat model of metabolic syndrome. *FEBS Letters*.

[48] Hyogo H, Tazuma S, Arihiro K (2008). Efficacy of atorvastatin for the treatment of nonalcoholic steatohepatitis with dyslipidemia. *Metabolism*.

[49] Kashi MR, Torres DM, Harrison SA (2008). Current and emerging therapies in nonalcoholic fatty liver disease. *Seminars in Liver Disease*.

[50] Nelson A, Torres DM, Morgan AE, Fincke C, Harrison SA (2009). A pilot study using simvastatin in the treatment of nonalcoholic steatohepatitis: a randomized placebo-controlled trial. *Journal of Clinical Gastroenterology*.

[51] Enjoji M, Nakamuta M (2010). Is the control of dietary cholesterol intake sufficiently effective to ameliorate nonalcoholic fatty liver disease?. *World Journal of Gastroenterology*.

